# Value of Patlak Ki images from ^18^F-FDG-PET/CT for evaluation of the relationships between disease activity and clinical events in cardiac sarcoidosis

**DOI:** 10.1038/s41598-021-82217-0

**Published:** 2021-02-01

**Authors:** Masatoyo Nakajo, Satoko Ojima, Hirofumi Kawakami, Atsushi Tani, Akira Hirayama, Megumi Jinguji, Takuro Kubozono, Mitsuru Ohishi, Takashi Yoshiura

**Affiliations:** 1 Department of Radiology, Kagoshima University, Graduate School of Medical and Dental Sciences, 8-35-1 Sakuragaoka, Kagoshima, 890-8544 Japan; 2 Department of Cardiovascular Medicine and Hypertension, Kagoshima University, Graduate School of Medical and Dental Sciences, 8-35-1 Sakuragaoka, Kagoshima, 890-8544 Japan; 3 Research and Development Department, GE Healthcare Japan, 4-7-127 Asahigaoka-Hinoshi, Tokyo, 191-8503 Japan

**Keywords:** Cardiology, Molecular medicine

## Abstract

The association between ^18^F-fluorodeoxyglucose (^18^F-FDG) myocardial uptake and clinical presentations in cardiac sarcoidosis (CS) has not yet been clarified. The Patlak slope, Ki*,* which represents the rate of ^18^F-FDG uptake is a quantitative index of ^18^F-FDG metabolism. This study aims to investigate the usefulness of standardized uptake value (SUV) and Patlak Ki images (Ki images) extracted from dynamic ^18^F-FDG-PET/CT for evaluating the risk of clinical events (CEs) in CS. The SUV and Ki myocardial images were generated from 30 dynamic ^18^F-FDG-PET/CT scans of 21 CS patients. The SUV and Ki images both were rated as positive in 19 scans and negative in 11 scans with the same incidence of CEs which were significantly higher in positive than negative scans [cardiac dysfunction: 78.9% (15/19) vs. 27.2% (3/11); arrhythmic events: 65.5% (10/19) vs. 0% (0/11)]. In 19 positive scans, the three Ki parameters (Ki max, Ki mean and Ki volume) were significantly higher in scans for patients with arrhythmic events than in those without. Logistic regression analysis showed that the Ki volume alone was significantly associated with the risk of arrhythmic events. Our study suggests that Ki images may add value to SUV images for evaluating the risk of CEs in CS patients.

## Introduction

Sarcoidosis is a systemic granulomatous disease of unknown etiology. Clinical manifestations of cardiac involvement are estimated to occur in 5% of patients with sarcoidosis, but the prevalence at autopsy has ranged from 25 to 58%^1,2^. Cardiac sarcoidosis (CS) has a poor prognosis because of frequent complication of atrioventricular block (AVB), ventricular tachycardia (VT) and congestive heart failure^3–5^. To prevent adverse outcomes, an accurate and early diagnosis is essential so that anti-inflammatory therapy can be initiated^6,7^.

Glucose metabolic activity can be shown by measuring ^18^F-fluorodeoxyglucose (^18^F-FDG) uptake during positron emission tomography (PET)/computed tomography (CT) for not only oncological but also inflammatory disorders^8,9^. Qualitative or quantitative ^18^F-FDG PET/CT has been used for diagnosing or assessing the disease activity of CS^10,11^. However, the association between ^18^F-FDG myocardial uptake and clinical presentations in CS has not yet been clarified.

The two most widely used quantitative indices of ^18^F-FDG metabolism are the standardized uptake value (SUV)^12,13^ and the Ki, which represents the rate of ^18^F-FDG uptake and is a quantitative index of ^18^F-FDG metabolism as measured by the Patlak slope^14,15^. SUV is a simple semiquantitative index, calculated by measuring the activity concentration in the lesion during a short-duration static scan acquired late (typically 60 min) after injection, and then normalized for the injected dose and either patient weight or lean body mass^16,17^. On the other hand, to calculate the Ki requires dynamic imaging to obtain an arterial input function (IF) and construct a lesion time-activity curve^14,15^. Thus, Patlak analysis is more demanding than calculation of SUV and inconvenient for patients. For these reasons, patient throughput can be much higher if SUV rather than Patlak analysis is used, accounting for the wide use of the SUV method.

Although a good correlation has been reported between the SUV and Ki, they are not equivalent^18^. The SUV measures the total activity in the lesion, and includes both metabolized ^18^F-FDG and unmetabolized ^18^F-FDG (unphosphorylated ^18^F-FDG) in the blood, intercellular spaces, and/or cells. Patlak analysis separates these two components, and the Patlak slope is determined only by metabolized ^18^F-FDG^13^. Thus, measurements of Ki might contribute to assessments of CS disease activity. However, to our knowledge, only one report has investigated dynamic ^18^F-FDG-PET/CT images for diagnosis of CS^19^, and it is unknown if Patlak Ki images (Ki images) extracted from dynamic ^18^F-FDG-PET/CT are useful for evaluating the disease activity or clinical events in CS patients.

The study aim was to investigate the usefulness of SUV and Ki images for evaluating the risk of clinical events (CEs) including cardiac dysfunction and arrhythmic events in CS patients.

## Results

### CEs at ^18^F-FDG PET/CT scan

The median left ventricular ejection fraction (LVEF) was 36.4% (interquartile range [IQR] 32.6–55.1%; range 23.5–79.9%). Cardiac dysfunction and normal cardiac function were observed in 18 and 12 ^18^F-FDG PET/CT scans, respectively. Of the 18 scans of patients with cardiac dysfunction, six and 12 were performed before treatment and under treatment, respectively. On the other hand, of the 12 scans of patients with normal cardiac function, two and ten were performed before treatment and under treatment, respectively. Arrhythmic events were observed for 10 ^18^F-FDG PET/CT scans, and seven and three scans were performed before treatment and under treatment, respectively. On the other hand, of 20 scans of patients without arrhythmic events, one and 19 were performed before treatment and under treatment, respectively.

### Relationships between visual findings of SUV or Ki images and CEs (Table 1)

**Table 1:** Relationships between visual SUV or Ki myocardial findings and clinical events in 30 ^18^F-FDG PET/CT Scans of 21 patients with cardiac sarcoidosis.

LVEF	Visual SUV and Ki image findings			
	Positive	Negative	P value	
Median	32.9%	56.3%	0.001	
IQR	30.38–43.75%	40.05–70.95%		
Range	23.5–58.8%	35.0–56.3%		

	Positive	Negative	Total	P value
**Cardiac function**				
Dysfunction (LVEF < 50%)	15	3	18	0.009
Normal (LVEF > 50%)	4	9	12	
Total	19	11	30	
**Arrhythmic events**				
Presence	10	0	10	0.004
Absence	9	11	20	
Total	19	11	30	

*LVEF* left ventricular ejection fraction, *IQR* interquartile range.

No differences were observed in positive or negative visibility among the 4 Ki images generated by 2 input ascending aorta ROIs set by 2 observers in all PET/CT examinations. The SUV and Ki mages were both rated as positive in 19 scans and negative in 11 scans, respectively, and for both the median LVEF was significantly lower in the positive images than in the negative images (positive vs. negative: 32.9% vs. 56.3%, p = 0.001). The rate of cardiac dysfunction was significantly higher in the positive images than in the negative images (positive vs. negative: 78.9% [15/19] vs. 27.2% [3/11], p = 0.009). The arrhythmic events were only observed in the positive images, and the rate of arrhythmic events was significantly higher in the positive images than in the negative images (positive vs. negative: 65.5% [10/19] vs. 0% [0/11], p = 0.004).

### Intra- and inter-observer variability

The intraclass correlation coefficient (ICC)s and Bland–Altman analysis for intra-observer variability for Ki parameters are summarized in supplemental Table S1. The ICCs were 0.99 for all Ki parameters [maximum Ki (Ki max), mean Ki (Ki mean), volume of Ki (Ki volume)] for each observer 1 or 2 indicating excellent agreement. Bland–Altman plots of the Ki max, Ki mean and Ki volume for each observer are presented in supplemental Figure S1. The mean difference and associated lower and upper reproducibility limits were − 0.6%, − 5.1% and + 4.0% for Ki max, − 0.4%, − 5.2% and + 4.4% for Ki mean, and 1.8%, − 7.2% and + 10.8% for Ki volume in observer 1, and − 0.3%, − 3.4% and + 2.7% for Ki max, 0.5%, − 4.6% and + 5.6% for Ki mean, and − 1.2%, − 12.3% and + 9.9% for Ki volume in observer 2, respectively.

Inter-observer agreement was excellent for all parameters between observers 1 and 2; ICCs for maximum SUV (SUVmax), mean SUV (SUVmean), cardiac metabolic volume (CMV), and cardiac metabolic activity (CMA) were 1.00 (95% confidence interval (CI) 1.00–1.00), 0.99 (95% CI 0.97–1.00), 0.97 (95% CI 0.92–0.98) and 0.99 (95% CI 0.97–1.00), and those for Ki max, Ki mean and Ki volume were 0.99 (95% CI 0.99–1.00), 0.99 (95% CI 0.99–1.00) and 0.99 (95% CI 0.97–1.00), respectively.

### Correlations between quantitative parameters

Quantitative analyses were performed in each positive SUV or Ki image (n = 19). Significant positive correlations were observed between SUVmax and Ki max (ρ = 0.88, p < 0.001), between SUVmean and Ki mean (ρ = 0.85, p < 0.001) and between CMV and Ki volume (ρ = 0.68, p = 0.001), respectively.

### Relationships between SUV or Ki parameters and CEs (Table 2)

**Table 2:** Relationships between quantitative SUV or Ki parameters and clinical events in 19 ^18^F-FDG positive myocardia patients.

Index	Cardiac function							Arrhythmic events						
	Normal (n = 4)			Dysfunction (n = 15)			p value	Absence (n = 9)			Presence (n = 10)			p value
	Median	IQR	Range	Median	IQR	Range		Median	IQR	Range	Median	IQR	Range	
SUVmax	3.91	2.68–6.03	2.47–7.12	3.99	3.08–7.52	2.91–11.69	0.37	3.15	2.97–4.23	2.89–5.82	6.18	3.54–8.51	2.47–11.69	0.060
SUVmean	2.71	2.43–3.65	2.24–4.50	3.10	2.68–4.29	2.54–6.84	0.27	2.69	2.60–3.08	2.54–3.29	3.92	2.72–4.66	2.24–6.84	0.041
CMV	8.70	3.78–32.4	2.6–52.4	44.0	11.6–130.8	5.6–223.0	0.072	15.9	7.40–30.9	5.0–83.7	96.2	12.5–164.5	2.6–223.0	0.066
CMA	20.4	12.3–87.3	11.7–146.7	119.5	30.8–664.5	15.1–1142.9	0.072	41.7	19.0–94.9	12.9–274.9	312.9	27.9–930.2	11.7–1142.9	0.060
Ki max (× 10^–3^)*	136.4	94.6–224.4	83.0–282.1	105.9	90.1–302.6	80.4–555.3	0.92	96.5	81.4–158.3	80.4–171.6	241.4	90.3–464.8	83.0–553.3	0.027
Ki mean (× 10^–3^)*	68.7	50.9–130.9	46.0–180.1	72.4	48.1–174.0	41.4–336.8	1.00	55.0	42.0–74.7	41.4–84.0	143.1	50.9–214.9	46.0–336.8	0.027
Ki volume^†^	15.4	8.65–46.5	2.7–6.7	43.1	14.45–100.8	7.8–115.3	0.27	14.6	12.9–20.0	7.8–33.0	82.8	43.3–106.2	2.7–115.3	0.003

*IQR* interquartile range, *CMV (cm3)* cardiac metabolic volume, *CMA* cardiac metabolic activity.

*The unit of Ki max and Ki mean are ml/g/min. ^†^The unit of Ki volume was cm^3^.

No significant differences between the scans of patients with cardiac dysfunction (n = 15) and normal cardiac function (n = 4) in the SUVmax and SUVmean were found (*p* > 0.05, each). The median CMV and CMA were higher in cardiac dysfunction than in normal cardiac function, but this difference was not significant (p = 0.072, both). No significant correlations were observed between any of the SUV parameters and LVEF (SUVmax, ρ = 0.018, p = 0.94; SUVmean, ρ = 0.035, p = 0.99; CMV, ρ = − 0.13, p = 0.60; CMA, ρ = − 0.13, p = 0.59, respectively). No significant differences in the Ki max, Ki mean and Ki volume between cardiac dysfunction and normal cardiac function were found (p > 0.05, each). No significant correlations were observed between each Ki parameter and LVEF (Ki max, ρ = 0.21, p = 0.40; Ki mean, ρ = 0.25, p = 0.29; Ki volume, ρ = − 0.14, p = 0.55).

The median SUVmean was significantly higher in the presence of arrhythmic events (n = 10) than in the absence of arrhythmic events (n = 9) (p = 0.041). Although the differences of SUVmax, CMA and CMV did not achieve statistical significance between the absence and presence of arrhythmic events (p = 0.060, 0.066, and 0.060, respectively), the median SUVmax, CMA and CMV were higher in the presence of arrhythmic events than in the absence of arrhythmic events, and these differences were close to a significance level (alpha = 0.05). On the other hand, although all three Ki parameters were significantly higher in the presence of arrhythmic events than in the absence of arrhythmic events, the differences of Ki max and Ki mean were close to a significance level (alpha = 0.05) (Ki max, 241.4 × 10^–3^ ml/g/min vs. 96.5 × 10^–3^ ml/g/min, p = 0.027; Ki mean, 143.1 × 10^–3^ ml/g/min vs. 55.0 × 10^–3^ ml/g/min, p = 0.027; Ki volume, 82.8 cm^3^ vs. 14.6 cm^3^, p = 0.003).

### Parameters for assessing the risk of CEs (Tables 3, 4)

**Table 3:** Ability of SUV and Ki parameters to discriminate between the presence and absence of clinical events.

Index	Cardiac dysfunction						Arrhythmic events					
	Cutoff value	Sensitivity	Specificity	Accuracy	AUC	p value	Cutoff Value	Sensitivity	Specificity	Accuracy	AUC	p value
SUVmax	> 2.89	80.0 (12/15) 51.9–95.7*	50.0 (2/4) 6.8–93.2*	73.7 (14/19) 48.8–90.9*	0.67 0.42–0.86*	0.41	> 5.82	50 (5/10) 18.7–81.3*	100 (9/9) 66.4–100*	73.7 (14/19) 48.8–90.9*	0.76 0.51–0.92*	0.034
SUVmean	> 2.61	86.7 (13/15) 59.5–98.3*	50.0 (2/4) 6.8–93.2*	78.9 (15/19) 54.4–93.9*	0.68 0.43–0.87*	0.30	> 3.29	60 (6/10) 26.2–87.8*	100 (9/9) 66.4–100*	78.9 (15/19) 54.4–93.9*	0.78 0.53–0.93*	0.018
CMV	> 4.95	100 (15/15) 78.2–100*	50.0 (2/4) 6.8–93.2*	89.5 (17/19) 66.9–98.7*	0.80 0.56–0.95*	0.031	> 83.7	50 (5/10) 18.7–81.3*	100 (9/9) 66.4–100*	73.7 (14/19) 48.8–90.9*	0.75 0.50–0.92*	0.040
CMA	> 12.9	100 (15/15) 78.2–100*	50.0 (2/4) 6.8–93.2*	89.5 (17/19) 66.9–98.7*	0.80 0.56–0.95*	0.031	> 274.9	50 (5/10) 18.7–81.3*	100 (9/9) 66.4–100*	73.7 (14/19) 48.8–90.9*	0.76 0.51–0.92*	0.034
Ki max^†^	< 105.9 × 10^–3^	53.3 (8/15) 26.6–78.7*	75.0 (3/4) 19.4–99.4*	57.9 (11/19) 33.5–79.7*	0.52 0.28–0.75*	0.91	> 171.6 × 10^–3^	60 (6/10) 26.2–87.8*	100 (9/9) 66.4–100*	78.9 (15/19) 54.4–93.9*	0.80 0.56–0.95*	0.005
Ki mean^†^	> 180.1 × 10^–3^	26.7 (4/15) 7.8–55.1*	100 (4/4) 39.8–100*	42.1 (8/19) 20.2–66.5*	0.50 0.27–0.73*	1.00	> 84.0 × 10^–3^	60 (6/10) 26.2–87.8*	100 (9/9) 66.4–100*	78.9 (15/19) 54.4–93.9*	0.80 0.56–0.95*	0.005
Ki volume^‡^	> 16.2	66.7 (10/15) 38.4–88.2*	75.0 (3/4) 19.4–99.4*	68.4 (13/19) 43.4–87.4*	0.68 0.43–0.87*	0.25	> 33.0	90 (9/10) 55.5–99.7*	100 (9/9) 66.4–100*	94.7 (18/19) 74.0–99.9*	0.90 0.68–0.99*	< 0.001

*CMV (cm3)* cardiac metabolic volume, *CMA* cardiac metabolic activity, *AUC* area under the receiver operating characteristic curve.

*95% confidence interval. ^†^The unit of Ki max and Ki mean are ml/g/min. ^‡^The unit of Ki volume was cm^3^.

**Table 4:** Logistic analysis for association between each parameter and presence of clinical events.

Index	Cardiac dysfunction				Arrhythmic events			
	*X*^2^	Odds ratio	95% CI	p value	*X*^2^	Odds ratio	95% CI	p value
SUVmax	0.48	1.20	0.72–1.98	0.49	3.31	1.81	0.95–3.44	0.069
SUVmean	0.55	1.67	0.43–6.49	0.46	3.10	5.06	0.83–30.83	0.078
CMV	1.33	1.03	0.98–1.08	0.25	3.43	1.02	0.99–1.05	0.064
CMA	0.94	1.01	0.99–1.03	0.33	2.26	1.01	0.99–1.01	0.13
Ki max	0.26	1.00	0.99–1.01	0.61	2.91	1.02	0.99–1.04	0.086
Ki mean	0.24	1.00	0.99–1.02	0.63	2.41	1.03	0.99–1.07	0.12
Ki volume	1.20	1.02	0.98–1.06	0.27	4.10	1.09	1.01–1.19	0.043

*CMV* cardiac metabolic volume, *CMA* cardiac metabolic activity, *CI* confidence interval.

The areas under the receiver operating characteristic curves (AUCs) for the ability to assess the risk of the cardiac dysfunction were 0.80 for CMV (p = 0.031) and 0.80 for CMA (p = 0.031) (Table 3). The logistic analysis revealed that no parameters were significantly associated with cardiac dysfunction (p > 0.05, each) (Table 4).

The AUCs for the ability to assess the risk of arrhythmic events were 0.76 for SUVmax (p = 0.034), 0.78 for SUVmean (p = 0.018), 0.75 for CMV (p = 0.040), 0.76 for CMA (p = 0.034), 0.80 for Ki max (p = 0.005), 0.80 for Ki mean (p = 0.005) and 0.90 for Ki volume (p < 0.001) (Table 3). The specificity was 100% for all parameters. The sensitivity ranged from 50.0% (SUVmax, CMV, CMA) to 90.0% (Ki volume), and the accuracy ranged from 73.7% (SUVmax, CMV, CMA) to 94.7% (Ki volume) (Table 3). Although no significant differences in AUC were found among any of the parameters (p > 0.05, each), only the Ki volume did not overlap in value between groups with and without arrhythmic events, except in one case, and resulted in the best diagnostic performance, with AUC of 0.90 and accuracy of 94.7%, as shown in the plot of each parameter in supplemental Figures S2 and S3. Moreover, the logistic analysis also revealed that Ki volume was the only parameter significantly associated with arrhythmic events (odds ratio 1.09, 95% CI 1.01–1.19, p = 0.043) (Table 4).

**Figure 1:**
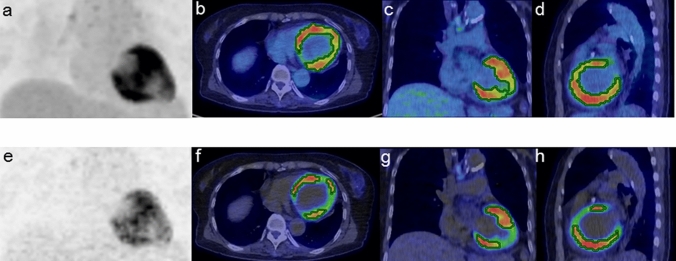
A 70-year-old female patient with CS who showed VT and cardiac dysfunction (EF: 40.0%) before treatment. The SUV images [maximum intensity projection (MIP) (**a**), transaxial (**b**), coronal (**c**), and sagittal (**d**)] and Ki images [MIP (**e**), transaxial (**f**), coronal (**g**), and sagittal (**h**)] both show the positive myocardium for which the VOIs were automatically determined. The SUV (SUVmax: 8.51 g/ml, SUVmean:4.66 g/ml, CMV: 220.0 cm^3^, CMA: 1025.2 g) and Ki parameters (Ki max: 370.2 × 10^–3^ ml/g/min, Ki mean: 214.9 × 10^–3^ ml/g/min, Ki volume: 115.3 cm^3^) were all positive for the risk of arrhythmic events according to each threshold criterion.

**Figure 2:**
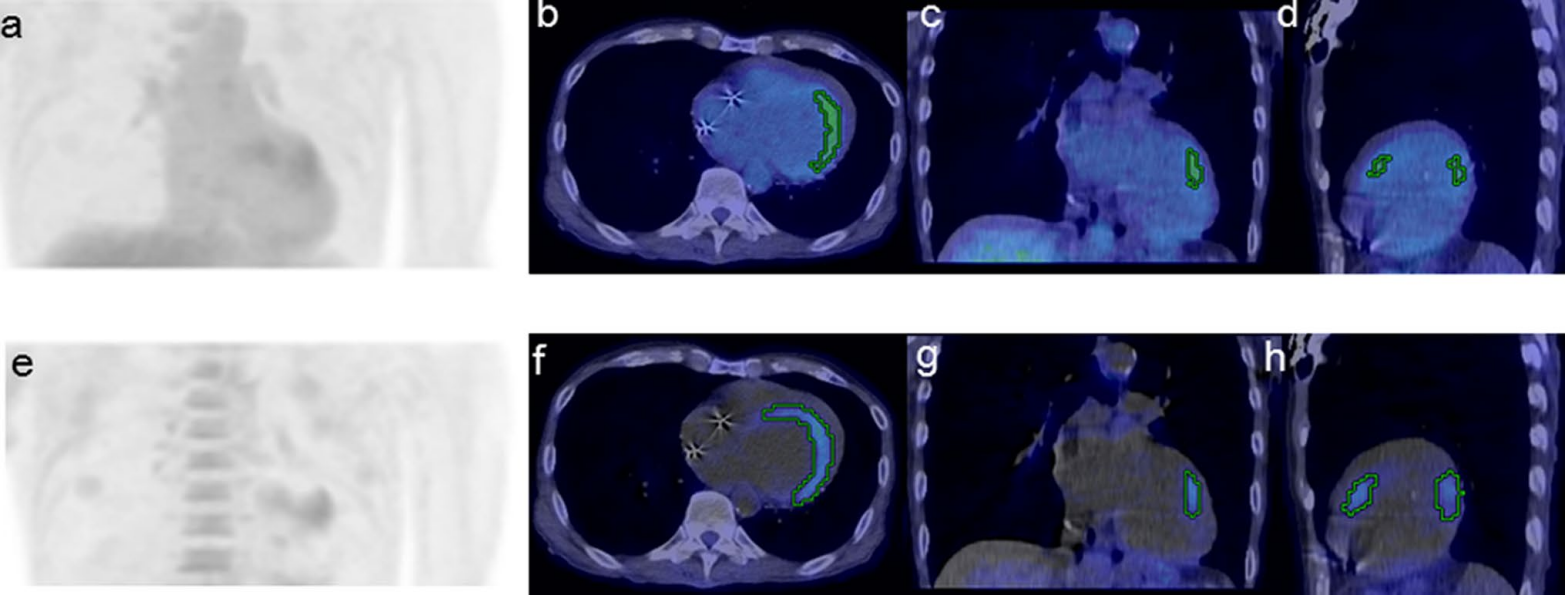
A 68-year-old male patient with CS who showed VT and cardiac dysfunction (EF: 25.3%) under steroid treatment (prednisolone, 10 mg daily). The SUV images [MIP (**a**), transaxial (**b**), coronal (**c**), and sagittal (**d**)] and Ki images [MIP (**e**), transaxial (**f**), coronal (**g**), and sagittal (**h**)] both show the positive myocardium for which the VOIs were automatically determined. The Ki volume (43.3 cm^3^) was the only parameter positive for the risk of arrhythmic events according to each threshold criterion.

**Figure 3:**
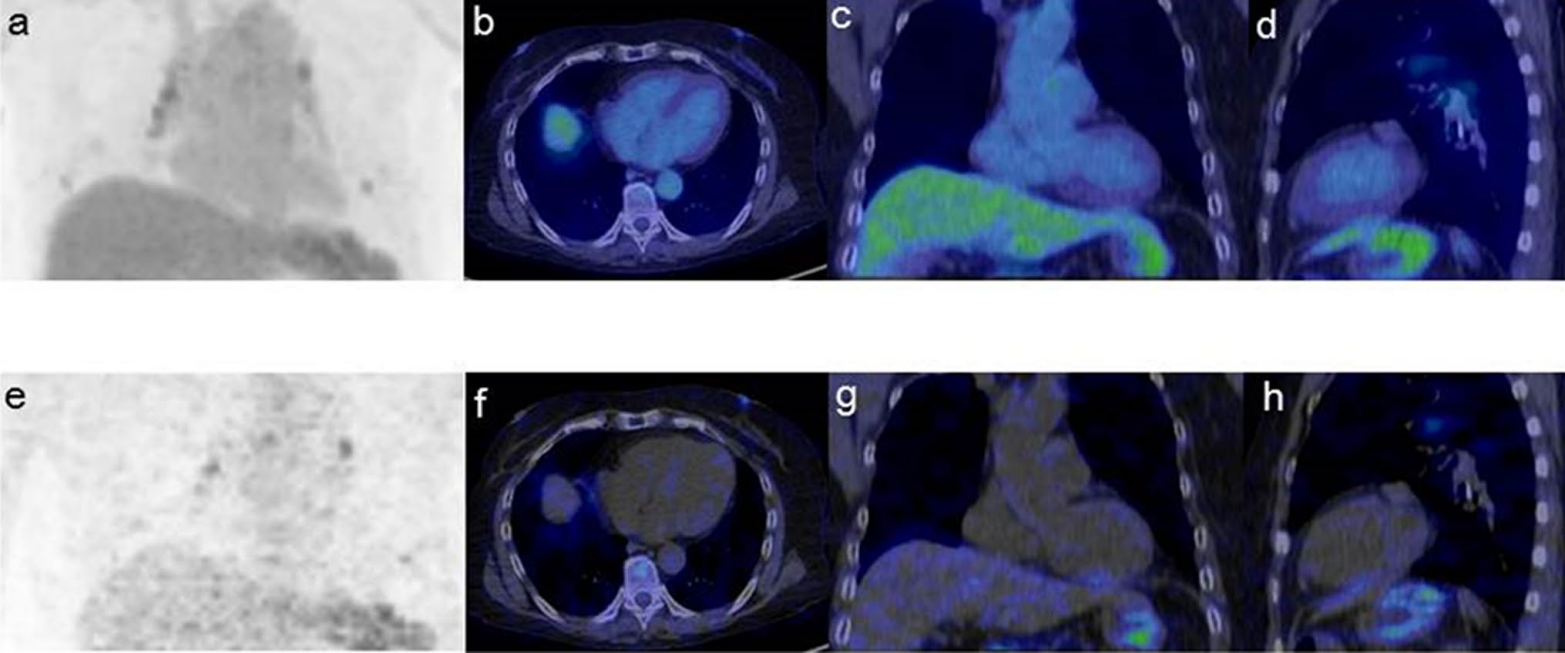
A 69-year-old female patient with CS who showed no arrhythmic events with normal cardiac function (EF: 70%) under steroid treatment (prednisolone, 10 mg daily). The SUV images [MIP (**a**), transaxial (**b**), coronal (**c**), and sagittal (**d**)] and Ki images [MIP (**e**), transaxial (**f**), coronal (**g**), and sagittal (**h**)] both show the negative myocardium.

The SUV and Ki images of the representative positive and negative scans of CS were shown in Figs. 1, 2 and 3.

## Discussion

Previous studies have reported that ^18^F-FDG SUV images have been important in the assessment of CS^10,11^. A recent meta-analysis reported a sensitivity of 89% and specificity of 78% for detection of CS on ^18^F-FDG SUV images^20^. The relationships between ^67^Ga scintigraphy or ^18^F-FDG SUV images and clinical events in CS patients have been reported previously^21–23^. Banba et al.^21^ performed ^67^Ga scintigraphy in 15 CS patients, and gallium uptake has been documented in 80% of AVB patients. McArdle et al.^22^ examined the degree of ^18^F-FDG myocardial uptake assessed by SUV parameters, and higher SUVmax and SUVmean were observed in CS patients with VT than in those with AVB or who were clinically silent. Ahmadian et al.^23^ reported that CMA calculated by the SUV threshold using the 1.5 times SUVmax of left ventricular blood pool was significantly higher in CS patients with an EF < 50% than in those with an EF > 50%, but SUVmax was not significantly different between them when only ^18^F-FDG positive cases were analyzed.

In our study, the rate of cardiac dysfunction or arrhythmic events was significantly higher in the visually analyzed positive SUV images than in the negative images, suggesting that visual assessment of SUV images might be useful for evaluating the risk of CEs. On the other hand, in quantitative analyses of the SUV positive scans, although each parameter was higher in the presence of cardiac dysfunction than in its absence and higher in the presence of arrhythmic events than in their absence, the difference was only significant for the SUVmean for arrhythmic events (p = 0.041) even if other SUV parameters were close to a significance level (alpha = 0.05). The discrepancy among the study results of SUV parameters for evaluating the clinical events may be due to the differences in the analysis methods. We performed the quantitative analyses only for visual ^18^F-FDG positive scans and chose an SUVmax threshold of 40% for CMV delineation, as was performed in a previous report^24^.

Dynamic PET imaging has been used in oncology for characterizing the kinetic FDG model^25,26^. Wang et al.^25^ reported a significant difference in both the SUVmax and Ki between benign and malignant pulmonary lesions with a high significant correlation between SUVmax and Ki and stated that parametric Ki images were useful for distinguishing malignant lesions from normal tissue.

We found only one study that used dynamic ^18^F-FDG-PET/CT images to diagnose CS^19^, and the researchers reported that heterogeneous myocardial glucose metabolism assessed by using a normalized coefficient of variation of the Ki could be useful for diagnosis of CS. However, no study has examined the relationships between Ki images and clinical events in CS patients.

In our study, on visual analyses of Ki images, the rate of cardiac dysfunction or arrhythmic events was significantly higher in the positive Ki scans than in the negative Ki scans, suggesting that the visual assessment of Ki images might be useful for evaluating the risk of CEs.

On quantitative analyses of Ki positive scans, although none of the Ki parameters was significantly different between cardiac dysfunction and normal cardiac function, they were significantly higher with the arrhythmic events than without them. Although both Ki max and Ki mean showed p values close to a significance level (alpha = 0.05) such as SUV parameters, Ki volume clearly differentiated the presence and absence of arrhythmic events without overlap of values, except for one case, and resulted in the best diagnostic performance. Moreover, Ki volume alone was significantly associated with arrhythmic events suggesting that Ki volume might be the most useful parameter for evaluating the risk of arrhythmic events. Despite the significant correlations between SUV and Ki parameters, there were discrepancies in the results of these parameters concerning the association with clinical events in CS. Thus, Ki images, especially the quantitative parameter of Ki volume may have the potential to provide additional value to the SUV images for evaluation of the clinical events in CS patients.

There were some study limitations that should be considered when interpreting our results. First, this was a retrospective study with a small sample. Therefore, a prospective study with a large sample is needed to confirm the validity of the present findings. Second, the different treatment statuses at the time of the dynamic ^18^F-FDG-PET/CT scans could have affected the positivity of the SUV and Ki images, which may have led to biased quantitative analysis. Third, we did not compare other useful imaging techniques, such as cardiac magnetic resonance imaging (MRI) or myocardial perfusion imaging with SUV or Ki images because only a minority of patients underwent cardiac MRI (n = 6) or myocardial perfusion imaging (n = 2). Forth, neither motion artifact correction nor partial volume correction was performed. The accuracy of IDIF will be affected by body motion and partial volume effects^27–29^. Thus, the influence of motion or partial-volume effects was not ignored for the quantitative analysis. van der Weerdt et al.^28^ examined which vascular structure is most suited for defining the IDIF in cardiac dynamic ^18^F-FDG scans, and reported that the use of the ascending aorta with a large ROI (diameter, approximately 15 mm) for defining the IF resulted in the best agreement with arterial blood sampling and suffered less from statistical noise. The study indicated that the ascending aorta is the best structure for defining IDIF without correction for partial volume effects. Lubberink et al.^30^ also reported that ascending aorta IDIFs can be used for Patlak analysis of cardiac ^18^F-FDG scans without further partial-volume correction of the ascending aorta data.

In our study, in an attempt to minimize partial volume effect, we chose the ascending aorta for the structure with a large ROI (diameter 15 mm) setting. Moreover, the image reconstruction was performed by a penalized likelihood algorithm (Q.Clear) with the semiconductor PET scanner which is less suffer from partial volume effects compared with the conventional OSEM algorithm^31,32^. Indeed, all Ki parameters exhibiting the high reproducibility with excellent inter- or intra-observer agreement. On the other hand, any correction will also lead to some degree of statistical degradation of signals^28^, and assessment of the various correction methods was beyond the scope of this study.

Fifth, the time window for the Patlak analysis could have affected the quantitative analysis^29^. In our study, we adopted the long time window (10–60 min) for the Patlak analysis according to the above mentioned study by van der Weerdt et al.^28^. A population-based IF (PBIF), which is a normalized average of measured arterial blood data from several subjects, is an alternative IF method^29^. The normalized PBIF by initial distribution volume (iDV) has been reported as having the following potential advantage; no subject to effects of body motion and partial volume effect, and the shortened protocol by only scan time for the Patlak analysis^29^. Thus, PBIF_iDV_ might have the potential to be a reasonable method with easy to perform the Patlak analysis in clinical routine practice. However, an ideal time window for the Patlak analysis is not clearly defined, and it is necessary to compare with the results of other time windows to confirm the validity of the present findings and to find the appropriate time window to evaluate the disease activity in CS patients. Finally, in the absence of guidelines on optimal reconstruction parameters for Ki images, we used different beta values of 350 and 700 for reconstruction of the SUV and Ki images, respectively. Further investigations of optimal reconstruction protocols for Ki imaging are warranted.

In conclusion, Ki images, especially the quantitative parameter of Ki volume may add value to SUV images for evaluating the risk of CEs in CS patients.

## Methods

### Study design and patient selection

This retrospective study was approved by the ethics committee on epidemiological studies, Kagoshima University Graduate School of Medical and Dental Sciences (No.190140), which waived the requirement for informed consent. This study was conducted in accordance with the Declaration of Helsinki and Ethical Guidelines for Medical and Health Research Involving Human Subjects. All methods were performed in accordance with the relevant guidelines and regulations. From April 2019 to January 2020, ^18^F-FDG-PET/CT was performed in 24 consecutive patients for suspected CS or known CS, and their clinical records were reviewed to identify patients for analysis. The inclusion criterion was diagnosis of CS according to the Japanese Society of Sarcoidosis and Other Granulomatous Disorders guidelines^33^. Patients with a history or coexistence of other cardiac disorders were excluded.

Two patients were excluded because of hypertrophic cardiomyopathy and one for insufficient evidence of CS. Finally, 21 CS patients were enrolled (14 women and seven men; mean ± standard deviation [SD], age 61 years ± 11; age range 37 − 76 years). Eight patients underwent ^18^F-FDG PET/CT scans before steroid treatment, and two of them underwent scans at the 6-months follow-up after initiation of steroid treatment. The remaining 13 patients received steroid therapy, and seven of them underwent follow-up scans during the study period. Consequently, 21 patients had a total of 30 ^18^F-FDG-PET/CT scans.

### Imaging protocols

All patients were instructed to fast for ≥ 18 h before PET/CT, which resulted in a mean plasma glucose level of 107 mg/dl (range 68–154 mg/dl) immediately before ^18^F-FDG intravenous injection.

All ^18^F-FDG PET/CT examinations were performed on a Discovery MI PET/CT (GE Healthcare, Milwaukee, WI). First, low-dose CT covering the entire heart was performed (slice thickness, 3.75 mm; pitch, 1.375 mm; 120 keV; auto mA [40–100 mA depending on patient body mass]; reconstructed matrix size, 512 × 512) with the transaxial and cranio-caudal field of view (FOV) of 70 cm and 20 cm that was used for attenuation correction of the PET images. Thereafter, ^18^F-FDG (227 MBq ± 28 [range 179–286 MBq]) was injected, and dynamic PET (single-bed) images covering the same cranio-caudal FOV as that of the above CT were acquired with the following PET frames. The acquisition began at the injection, with scan times of 10 s/frame for the first 2 min, 3 min/frame for the next one frame, and 5 min/frame thereafter for a total of 60 min. The motion corrections including body motion and respiratory motion were not performed for the dynamic PET data. The dynamic data were reconstructed by using a matrix size of 128 × 128 with a Bayesian penalized likelihood (BPL) reconstruction algorithm^31^ (with point spread function incorporated as a default setting (Q.Clear, GE Healthcare, Milwaukee, WI) with a beta value (penalization factor) of 700 and transaxial FOV of 50 cm. The PET transaxial spatial resolution was 3.9 mm full-width half-maximum (FWHM) in-plane^32^. The registration of CT and reconstructed dynamic PET image was verified using ACQC (Attenuation Correction Quality Control, GE Healthcare) software on the PET/CT scanner.

### Generation of Ki and SUV static images

To determine the ^18^F-FDG kinetic parameters within the lesion, a linear approximation of the mathematical representation of the standard two-compartmental model with irreversible trapping was used according to Patlak analysis^14^. From C_i_ (tk), the ^18^F-FDG activity concentration in the lesion (Bq mL_tissue_^−1^) at a given time tk after injection, the analytical solution of the two-compartment model is given as:

$$ {\text{Ci }}\left( {{\text{tk}}} \right) = {\text{Ki}}\mathop \smallint \limits_{0}^{tk} {\text{Cp}}\left( {\text{t}} \right){\text{dt}} + {\text{VpCp}}\left( {{\text{tk}}} \right) $$

where Cp(tk) represents the ^18^F-FDG activity concentration in blood plasma at time tk (Bq mL_blood_^−1^) and Vp is the total blood distribution volume (i.e., the unmetabolized fraction of ^18^F-FDG in blood and interstitial volume).

The compartmental transfer rates, *K*1 (from blood to cell), *k*2 (from cell to blood), and *k*3 (from ^18^F-FDG to ^18^F-FDG-6-phophate), were used to calculate Ki, the net influx rate, as follows:

$$ {\text{Ki }} = \, \left( {K{\text{1 x}}k{3}} \right) \, / \, \left( {k{2 } + k{3}} \right). $$

The transfer rate *k*4 from ^18^F-FDG-6-phosphate to ^18^F-FDG is negligible because the Patlak analysis assumes unidirectional uptake of ^18^F-FDG (k4 = 0). The Ki unit is ml/g/min.

In this study, the non-invasive plasma arterial IF estimation technique using a Patlak graphical plot method was applied to calculate the Ki for generating the Ki images^14^. We characterized the IF by using blood time-activity curves derived from PET image (Image-derived input functions [IDIFs]). The following region of interest (ROI) setting was performed to determine the arterial IF by two radiologists independently. Both investigators were aware of the study purpose but blinded to clinical information. A 15-mm diameter spherical ROI was manually drawn in the center of the ascending aorta on the registered image to reduce the contamination such as atherosclerotic plaques or smooth muscles in the arterial wall. To avoid the bias of statistical noise, two ROIs were placed over two consecutive slices. Thus, 2 IDIFs were generated by each investigator and a total 4 IDIFs were generated for each study. The Patlak analysis was performed over the period from 10 to 60 min after injection during a steady state. The data were reconstructed by using a matrix size of 128 × 128 with a BPL reconstruction algorithm with a beta value of 700 and transaxial FOV of 50 cm as the Ki image. Two Ki images were created using each generated IDIF data by each investigator, thus a total of 4 Ki images were created for each study.

The PET frames acquired 53–60 min after ^18^F-FDG injection were corrected for attenuation by using the CT data, and these attenuation corrected images were reconstructed by using a matrix size of 128 × 128 with a BPL reconstruction algorithm with a beta value of 350 and transaxial FOV of 50 cm as the static SUV image (SUV image).

### Image analysis

Two nuclear medicine radiologists, who were aware of the study purpose but blinded to clinical information, interpreted the SUV and Ki images (4 Ki images for each study) together to reach in a consensus. First, the radiologists visually scored the ^18^F-FDG myocardial uptake by using a five-point scale for each SUV image as follows, 0, no visible uptake; (1) ^18^F-FDG uptake equal to blood pool (of the descending aorta); (2) higher than blood pool but lower than hepatic uptake; (3) somewhat higher than hepatic uptake; and (4) noticeably higher than hepatic uptake^24,34^. To determine the disease activity of CS, scores of 0–1 and 2–4 were assigned as negative and positive, respectively. Thereafter, they visually assessed each Ki image as negative (myocardial visibility was lower than or similar to that of liver) or positive (myocardial visibility was higher than that of liver).

The following quantitative parameters were obtained in the interpreted positive (visible) myocardium by the above 2 radiologists who were generated IDIFs; the SUVmax, SUVmean, CMV, and CMA for SUV images, and Ki max and Ki mean and Ki volume for Ki images. Each observer set the volumes of interest (VOIs) separately for SUV images and Ki images independently. They placed the VOIs manually on a suitable reference fused axial image and then defined the cranio-caudal and mediolateral extent encompassing the entire positive myocardial lesion, excluding any avid extracardiac structures, to obtain SUVmax and Ki max. They next set a 40% threshold of SUVmax^35^ or of Ki max to automatically delineate a VOI equal to or greater than the 40% threshold of SUVmax or Ki max to calculate the SUVmean, CMV, and CMA or Ki mean and Ki volume, respectively. On the quantitative analysis for Ki images, the above VOI setting was performed for each 2 Ki images. The measured values for each SUV-related or Ki-related parameter were averaged to represent the results of each parameter. The CMA was defined as the SUVmean x CMV. Workstations (Xeleris or Advantage Windows Workstation 4.5; GE Healthcare) calculated the SUVmax, SUVmean, CMV, CMA and the Ki max, Ki mean and Ki volume automatically.

### Ascertainment of CEs

Echocardiography was performed within 1 month of the ^18^F-FDG-PET/CT (mean ± SD, 6 days ± 8; range − 26 to + 26 days), and the echocardiography report was used as the reference standard for cardiac function. Cardiac dysfunction was defined as a LVEF < 50%^36^. Patients were also assessed to determine if arrhythmic events, including sustained VT or AVB were presented within 1 month of the ^18^F-FDG-PET/CT (mean ± SD, 8 days ± 8; range − 24 to + 31 days). AVB was characterized as either second- or third-degree AVB or trifascicular block documented on 12-lead or Holter echocardiography^36,37^.

### Statistical analysis

The agreement between the results (intra- and inter-observer variability) was measured using ICC and Bland and Altman analysis. The ICC is reported with a 95% confidence interval where ICC < 0.4 was considered poor agreement, ICC 0.40–0.59 was considered fair agreement, ICC 0.60–0.74 was considered good agreement, and ICC > 0.74 was considered excellent agreement^38^. Bland–Altman analyses were expressed as the mean difference and 95% limits of agreement^39^. Lower and upper reproducibility limits, defining the reference range of spontaneous changes, were calculated as ± 1.96 × SD, which provided that the distribution was not statistically different from a normal one.

The incidence of each CE was compared between the positive and negative images by using Fisher’s exact test. Quantitative variables were compared between scans with and without CEs by using the Mann–Whitney *U* test. The Spearman rank correlation was used to assess the relationship between two quantitative variables. Receiver operating characteristic (ROC) curve analysis was performed to examine the diagnostic performance of each parameter between the presence and absence of CEs, and the Youden index was used to determine the best cutoff point for each parameter^40^. The statistical significance of differences between the AUCs were analyzed by using the DeLong method^41^. The association between each parameter and presence of CEs was analyzed by logistic regression analysis.

Data are presented as medians and IQRs. A value of p < 0.05 was considered to be indicative of statistical significance, and all *p* values were two-tailed. MedCalc Statistical Software (MedCalc Software Ltd, Acacialaan 22, 8400 Ostend, Belgium) was used for the statistical analyses.

## Supplementary Information

Supplementary Information

## Data Availability

The datasets used and/or analyzed during the current study are available from the corresponding author on reasonable request.
