# Supplementary material for: Value of Patlak Ki images from 18F-FDG-PET/CT for evaluation of the relationships between disease activity and clinical events in cardiac sarcoidosis
Source: Sci Rep. 2021 Feb 1;11:2729. doi: 10.1038/s41598-021-82217-0 (PMC7851386; doi:10.1038/s41598-021-82217-0)
Supplement: Supplementary file 1 — Supplementary Information [file 41598_2021_82217_MOESM1_ESM.docx]

**Value of Patlak Ki images from ^18^F-FDG-PET/CT for evaluation of the relationships between disease activity and clinical events in cardiac sarcoidosis**

Masatoyo Nakajo^1*^, Satoko Ojima^2^, Hirofumi Kawakami^3^, Atsushi Tani^1^, Akira Hirayama^3^, Megumi Jinguji^1^, Takuro Kubozono ^2^, Mitsuru Ohishi^2^, Takashi Yoshiura^1^

^1^Department of Radiology, Kagoshima University, Graduate School of Medical and Dental Sciences, 8-35-1 Sakuragaoka, Kagoshima 890-8544, Japan.

^2^Department of Cardiovascular Medicine and Hypertension, Kagoshima University, Graduate School of Medical and Dental Sciences, 8-35-1 Sakuragaoka, Kagoshima 890-8544, Japan.

^3^Research and Development Department, GE Healthcare Japan, 4-7-127 Asahigaoka-Hinoshi, Tokyo 191-8503, Japan.

Masatoyo Nakajo

Department of Radiology, Kagoshima University, Graduate School of Medical and Dental Sciences;

E-mail: [toyo.nakajo@dolphin.ocn.ne.jp](mailto:toyo.nakajo@dolphin.ocn.ne.jp)

Satoko Ojima

Department of Cardiovascular Medicine and Hypertension, Kagoshima University, Graduate School of Medical and Dental Sciences; E-mail: [satoko9cats@gmail.com](mailto:satoko9cats@gmail.com)

Hirofumi Kawakami

Research and Development Department, GE Healthcare Japan; E-mail: [Hirofumi.Kawakami@ge.com](mailto:Hirofumi.Kawakami@ge.com)

Atsushi Tani

Department of Radiology, Kagoshima University, Graduate School of Medical and Dental Sciences;

E-mail: [atsutani3of@hotmail.com](mailto:atsutani3of@hotmail.com)

Akira Hirayama

Research and Development Department, GE Healthcare Japan; E-mail: [akira.hirayama@ge.com](mailto:akira.hirayama@ge.com)

Megumi Jinguji

Department of Radiology, Kagoshima University, Graduate School of Medical and Dental Sciences; E-mail: [jinmegu@gmail.com](mailto:jinmegu@gmail.com)

Takuro Kubozono

Department of Cardiovascular Medicine and Hypertension, Kagoshima University, Graduate School of Medical and Dental Sciences; E-mail: [kubozono@m.kufm.kagoshima-u.ac.jp](mailto:kubozono@m.kufm.kagoshima-u.ac.jp)

Mitsuru Ohishi,

Department of Cardiovascular Medicine and Hypertension, Kagoshima University, Graduate School of Medical and Dental Sciences; E-mail: [ohishi@m2.kufm.kagoshima-u.ac.jp](mailto:ohishi@m2.kufm.kagoshima-u.ac.jp)

Takashi Yoshiura

Department of Radiology, Kagoshima University, Graduate School of Medical and Dental Sciences; E-mail: [yoshiura@m3.kufm.kagoshima-u.ac.jp](mailto:yoshiura@m3.kufm.kagoshima-u.ac.jp)

^*^Correspondence to: Masatoyo Nakajo, MD, PhD,

Department of Radiology, Kagoshima University, Graduate School of Medical and Dental Sciences, 8-35-1 Sakuragaoka, Kagoshima 890-8544, Japan.

Tel: +81-99-275-5417, FAX: +81-99-265-1106,

E-mail: [toyo.nakajo@dolphin.ocn.ne.jp](mailto:toyo.nakajo@dolphin.ocn.ne.jp)

**
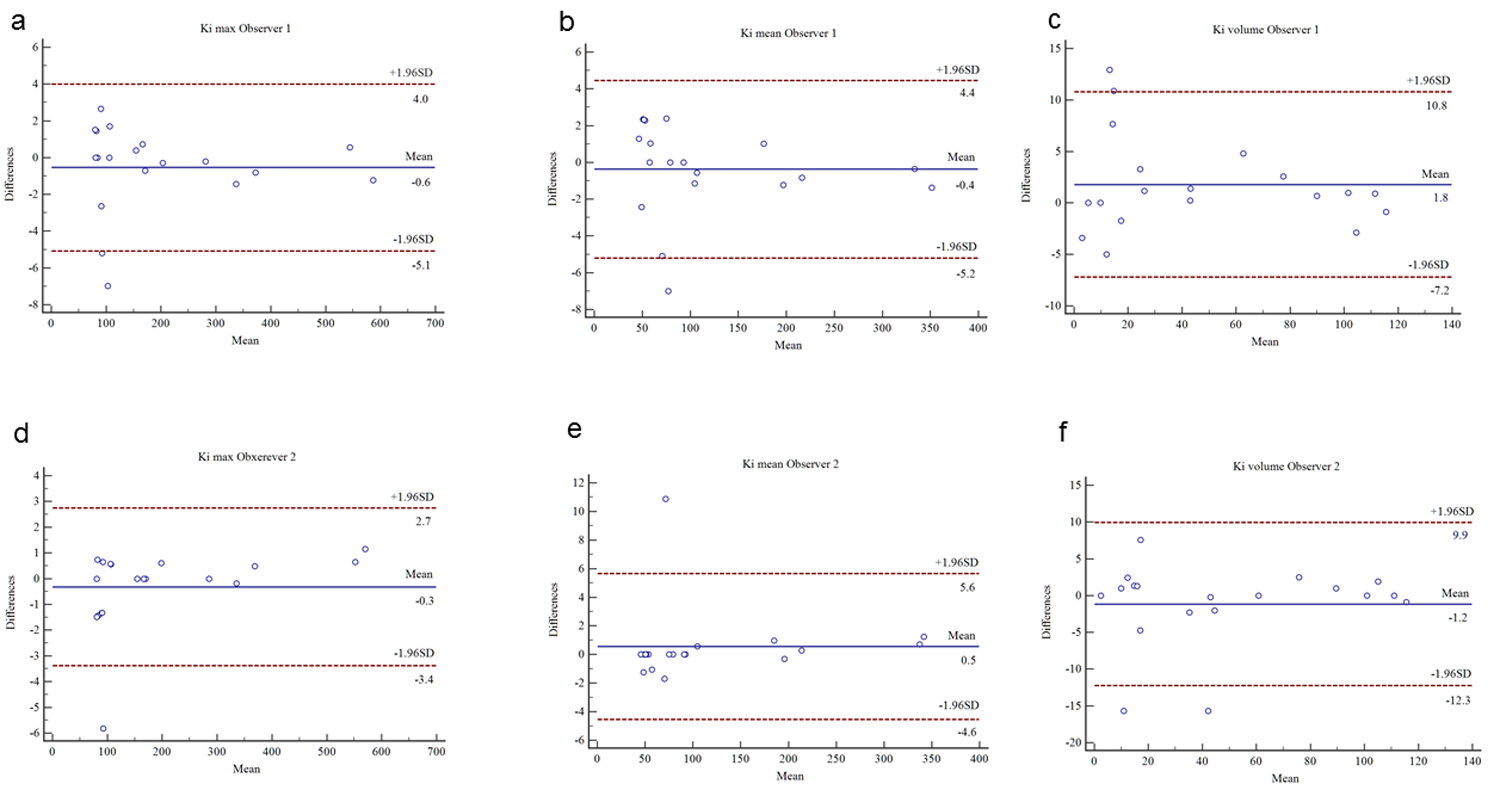
**

**Supplemental figure 1.** Intra-observer Bland-Altman plots of Ki parameters [Ki max (a, d), Ki mean (b, e), and Ki volume (c, f))]. Bland-Altman plots were used to analyze the agreement between the two evaluations per observer. The difference between two evaluations of one observer was plotted on the vertical axis and the mean of the two evaluations was plotted on the horizontal axis. The solid line represents the mean value for the data points and the dashed line represents the 1.96 × SD.


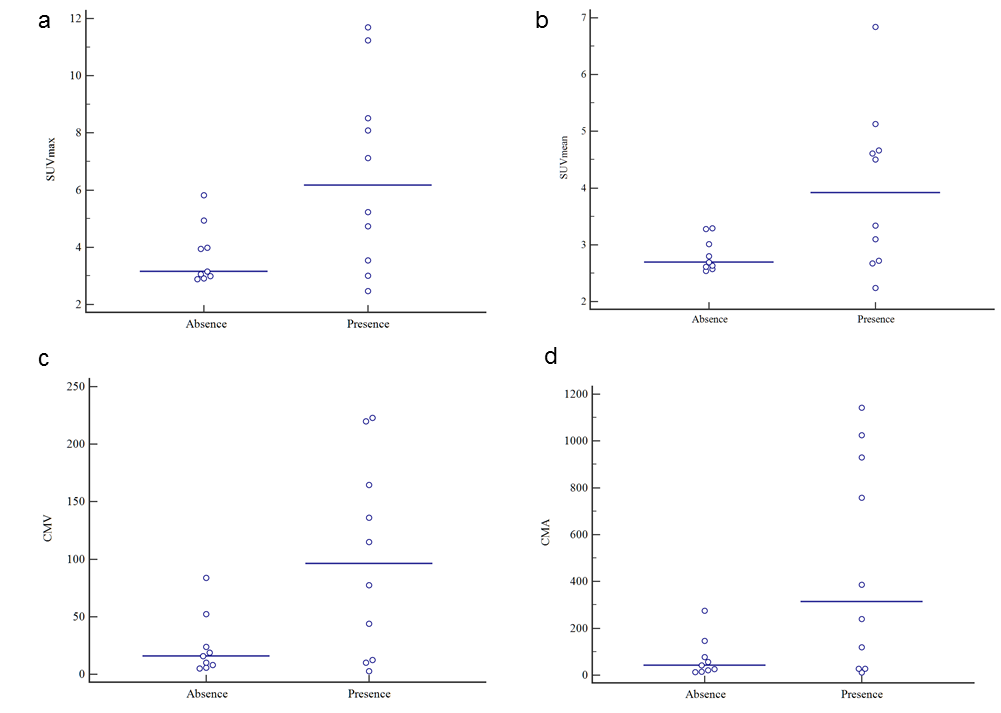


**Supplemental figure 2.** The plots of SUVmax (a), SUVmean (b), CMV (c), and CMA (d). Each parameter shows the overlap between the absence and presence of arrhythmic events.


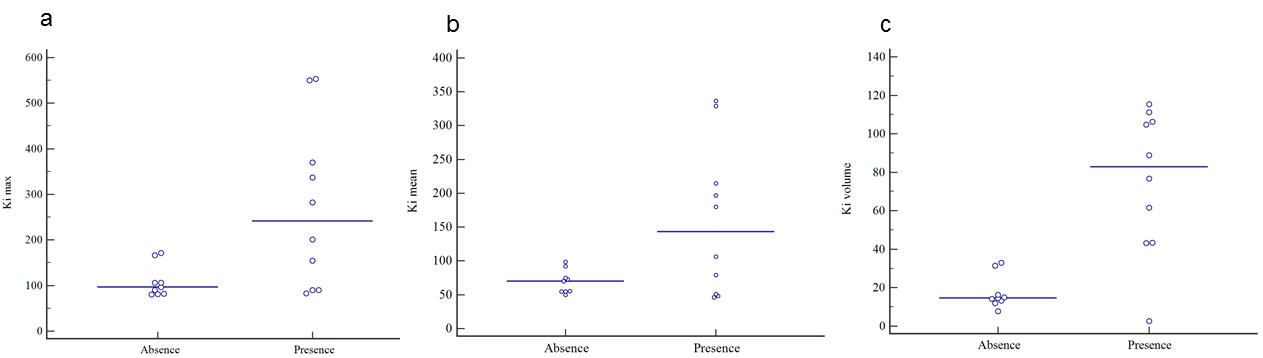


**Supplemental figure 3.** The plots of Ki max (a), Ki mean (b), and Ki volume (c). Ki max and Ki mean show the overlap between the absence and presence of arrhythmic events. Ki volume clearly differentiates the absence and presence of arrhythmic events without overlap of values, except for one case.

|  | **Observer 1** | | **Observer 2** | |
| --- | --- | --- | --- | --- |
|  | Intraclass correlation coefficient^*^ | Bland-Altman analysis^†^ | Intraclass correlation coefficient^*^ | Bland-Altman analysis^†^ |
| Ki max | 0.99 (0.99-1.000) | -0.6% (-5.1%, +4.0%) | 0.99 (0.99-1.000) | -0.3% (-3.4%, +2.7%) |
| Ki mean | 0.99 (0.99-1.000) | -0.4% (-5.2%, +4.4%) | 0.99 (0.99-1.000) | 0.5% (-4.6%, +5.6%) |
| Ki volume | 0.99 (0.99-0.99) | 1.8% (-7.2%, 10.8%) | 0.99 (0.99-0.99) | -1.2% (-12.3%, +9.9%) |

**Supplemental table 1.** Intra-observer agreement for Ki parameters with intraclass correlation coefficient and Bland-Altman analysis.

^*^ Numbers in parentheses are 95 % confidence intervals. ^†^ The mean difference and in parentheses 95 % limits of agreement are shown.
